# Triglyceride–glucose index and FIB-4 score in relation to cardiovascular disease risk among people with HIV: a retrospective cohort study

**DOI:** 10.3389/fmed.2025.1638071

**Published:** 2025-11-06

**Authors:** María Luisa Montes, Carmen Busca, Juan Martín-Torres, José Ignacio Bernardino, Francisco Arnaiz de las Revillas, Luz Martín-Carbonero, Jorge Sánchez Villegas, Rafael Micán, David Dalmau, Maria Mar Arcos, María de la Villa López Sánchez, Alejandro de Gea, Sofía Ibarra Ugarte, José Ramón Arribas, Juan González-García

**Affiliations:** 1 Unidad VIH, Servicio de Medicina Interna, Hospital Universitario La Paz, IdiPAZ, Madrid, Spain; 2 CIBERINFEC, Carlos III Health Institute, Madrid, Spain; 3 Servicio de Medicina Interna, Unidad VIH, Hospital Universitario 12 de Octubre, Madrid, Spain; 4 Servicio de Enfermedades Infecciosas, Hospital Universitario Marqués de Valdecilla Universidad de Cantabria, IDIVAL, Santander, Spain; 5 Enfermedades Infecciosas, Hospital Universitario Virgen de la Macarena, Seville, Spain; 6 Unidad VIH/ITS/PrEP Hospital Universitari Mutua Terrassa, Universitat de Barcelona, Barcelona, Spain; 7 Unidad de Enfermedades Infecciosas, Hospital Universitario de Jaén, Jaén, Spain; 8 Hospital Universitario Basurto, Bilbao, Spain

**Keywords:** HIV, cardiovascular events, hepatic steatosis, liver fibrosis, MASLD

## Abstract

**Background:**

People with HIV (PWH) have a high risk of cardiovascular events (CVEs). We investigated the incidence of CVEs in PWH and the usefulness of combining hepatic steatosis/insulin resistance (HS-IR) and risk of liver fibrosis for the evaluation of cardiovascular risk in PWH.

**Methods:**

We retrospectively analyzed 7,286 PWH from the prospective CoRIS cohort. We calculated the baseline triglyceride-glucose index (TyG) and FIB-4 index to assess HS-IR and risk of fibrosis, respectively, and evaluated persons with abnormal values for both indices. The primary outcome was the incidence of CVEs, defined as myocardial infarction, coronary disease, stroke, transient ischemic attack, peripheral arterial obstruction, and/or cardiovascular death. The association between HS-IR and risk of fibrosis and incidence of CVEs was assessed using a univariable and multivariable competing risk survival regression analysis.

**Results:**

The overall incidence of CVEs was 3.5 per 1,000 person-years. HS-IR and risk of fibrosis were significantly associated with an increased risk of CVEs. Individuals with HS-IR and risk of fibrosis experienced markedly more CVEs than those with normal values (10.6 vs. 1.4 per 1,000 person-years, *p* < 0.001). After correction for possible confounders and traditional cardiovascular risk factors, abnormal values for HS-IR and risk of fibrosis score were independently associated with CVEs of (HR, 2.21 [1.2–4.1]; *p* < 0.01).

**Conclusion:**

HS-IR and risk of fibrosis before ART are associated with increased risk of CVEs in PWH. A combined risk assessment incorporating HS-IR and risk of fibrosis may improve cardiovascular risk stratification in this population. These readily accessible tools can facilitate early identification and intervention in high-risk individuals.

## Highlights

- Hepatic steatosis, insulin resistance, and liver fibrosis predict cardiovascular events in PWH.
- A combined hepatic steatosis–insulin resistance and fibrosis score significantly improves cardiovascular risk assessment.
- Simple clinical indices enable early identification and targeted interventions for high-risk PWH.

## 1 Introduction

People with HIV (PWH) face a higher risk of cardiovascular diseases than the general population (1) because of factors such as the direct effects of the virus, adverse effects of antiretroviral therapy (ART), chronic inflammation, and comorbidities (e.g., metabolic disorders, arterial hypertension, and diabetes) (2). Hepatic steatosis (HS) and fibrosis, both of which are common in HIV infection, have also emerged as risk factors. Globally, the burden of HIV-related cardiovascular disease has tripled in the past 2 decades, mainly in Sub-Saharan Africa and Asia Pacific (1, 3). This seems to be because the PWH population is aging and because of general worsening of metabolic factors, even in developing areas such as Africa or Asia. Consequently, we must seek to better understand the drivers of increased risk of cardiovascular disease, including the role of hepatic conditions, in order to develop targeted interventions and improve cardiovascular health in PWH.

Hepatic steatosis and fibrosis in patients with fatty liver disease, but not viral hepatitis, are common in PWH, affecting 50 and 30%, respectively (4, 5). These conditions are often linked to metabolic disorders, chronic inflammation, and specific ART regimens, including d-nucleoside analogs and first-generation protease inhibitors. HS is associated with endothelial dysfunction and increased arterial inflammation, providing plausible biological mechanisms for its link to atherosclerotic cardiovascular disease (1, 6). HIV infection itself may contribute directly to the development of HS and fibrosis, thus potentially increasing the risk of cardiovascular events (CVEs) (7, 8). Similarly, hepatic fibrosis has been linked to increased cardiovascular risk, potentially through its effects on vascular structure and function.

The triglyceride-glucose (TyG) index is a simple, non-invasive, and inexpensive tool that can be used to estimate insulin resistance (IR), which is a hallmark of metabolic dysfunction–associated steatotic liver disease (MASLD) (9). It correlates well with the gold standard method of measuring IR, the hyperinsulinemic-euglycemic clamp, and with hepatic steatosis (10). Therefore, the index can be a useful marker for identifying MASLD even in PWH (11–13). The risk of liver fibrosis in PWH can also be assessed using the non-invasive Fibrosis 4 (FIB-4) index, which has been validated in individuals with HIV-HCV coinfection and MASLD and is a reliable predictor of CVEs in this population (5, 14, 15). In 2022, Martínez-Urbistondo et al. (16) reported the ability of the combination of TyG and FIB-4 to predict major cardiovascular events in European patients aged >40 years, independently of classic cardiovascular risk factors. By incorporating both indices, the TyG index and the FIB-4 index, as markers of HS-IR and risk of fibrosis into the clinical management of PWH, healthcare providers can gain a more comprehensive understanding of patients’ metabolic and hepatic status, thus making it easier to identify those at increased risk of CVEs and guide the implementation of earlier interventions to improve overall health outcomes (17).

The objective of this study was to evaluate whether markers of IR (TyG) and liver fibrosis risk (FIB-4 index), either alone or in combination, are associated with the incidence of cardiovascular disease in a prospective cohort of PWH and whether this association is independent of traditional cardiovascular risk factors.

## 2 Materials and methods

### 2.1 Study design and setting

Ours was a retrospective observational study based on data collected prospectively from the Cohort of the Spanish AIDS Research Network (in Spanish, Cohorte de la red española de investigación en sida [CoRIS]), a multicenter prospective cohort of PWH aged ≥13 years from 47 participating centers in 13 of the 17 autonomous communities in Spain. More detailed information can be found in Sobrino-Vegas et al. (18). Patients were ART-naïve at study entry. The CoRIS database collects baseline and follow-up sociodemographic, immunological, and clinical data, HIV transmission category, history of ART, previous opportunistic diseases, specific non-AIDS diseases, and serological and immunovirological data. Since 2014, height and weight have been recorded regularly at recruitment; weight is also recorded at follow-up visits. Data are organized and standardized following the HIV Cohorts Data Exchange Protocol (HICDEP) for data collection^1^ and adhere to internal strict annual quality controls. Patients are followed periodically according to routine clinical practice.

### 2.2 Study participants

**FIGURE 1:**
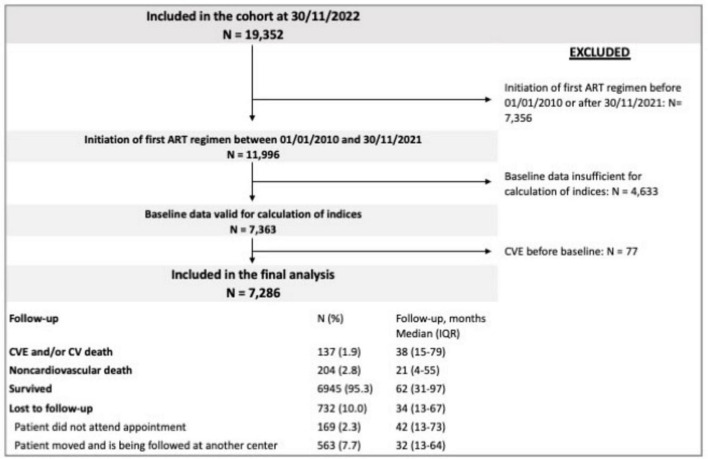
Flow diagram showing patients included in the study and in the final analysis. The reasons for loss to follow-up are specified.

On November 30, 2022, data from 19,352 PWH were registered in the CoRIS database. Of these, 7,286 met the inclusion criteria. For participants enrolled in the cohort, updated clinical and biological data were requested at 6 ± 2 months. Newly diagnosed cardiovascular comorbidities are specifically recorded following a well-defined protocol, according to the International Classification of Diseases Tenth Revision (ICD-10) (19). Losses to follow-up are shown in Figure 1.

Participants were followed up from initiation of ART until diagnosis of a CVE, death, transfer to another follow-up center, or failure to attend scheduled visits. ART regimens, occurrence of non-AIDS events, concomitant medication, and laboratory parameters were assessed at baseline and at the 6-month visit. Each CoRIS participant provided his or her written informed consent prior to enrolling in this study. The CoRIS cohort was approved by the Research Ethics Committee of Gregorio Marañón Hospital. This study was approved by Ethics Committee of Hospital Universitario La Paz, Madrid, Spain (HULP: PI-3706).

### 2.3 Inclusion and exclusion criteria

To be eligible for this study, participants had to be PWH aged ≥18 years who initiated ART as of January 1, 2010 and had not been diagnosed with CVEs (defined as myocardial infarct, coronary disease, stroke, transient ischemic attack, peripheral arterial obstruction, and/or cardiovascular death).

Patients with missing variables concerning the calculation of TyG or FIB-4 indices at baseline were excluded.

### 2.4 Variables analyzed

Sociodemographic, epidemiological, anthropometric, clinical, analytical, and therapeutic variables were analyzed before initiation of ART. The non-invasive TyG index {*Ln* [fasting triglycerides (mg/dL) × fasting glucose (mg/dL)]/2} was used to identify patients with HS-IR and a high probability of developing metabolic syndrome with a cut-off of >8.38 for HS-IR. Risk of liver fibrosis was assessed based on the FIB-4 index, with a cut-off of >1.3 as previously described (11, 20).

The cardiovascular risk factors analyzed at baseline were smoking, arterial hypertension, diabetes, hypercholesterolemia (total cholesterol >200 mg/dL), and treatments for arterial hypertension, diabetes, or dyslipidemia (therapy with statins and/or fibrates). Alcohol consumption was also analyzed based on standard drinks per week, with high-risk intake defined as >28 per week for men and >17 per week for women (21).

### 2.5 Primary outcome measure

The main outcome measure was a diagnosis of a CVE after initiation of ART made by the attending physician according to currently accepted definitions (19).

### 2.6 Independent variables

The covariates analyzed included sex at birth, age, baseline CD4 + T-cell count, months with HIV, prior AIDS-defining conditions, HCV coinfection, TyG and FIB-4 indices at baseline before initiation of antiretroviral treatment, and number of cardiovascular risk factors.

### 2.7 Statistical analysis

We reported the sociodemographic and clinical characteristics of the study population at initiation of ART using frequency tables for categorical variables and median and interquartile range for continuous variables. Groups were compared using the χ^2^ test and Fisher exact test (with the Freeman-Halton extension) in the case of categorical variables and the Mann-Whitney test or Kruskal-Wallis 1-way ANOVA (with multiple pairwise comparisons) in the case of quantitative data.

The incidence of new diagnoses of CVEs (per 1,000 person-years) was calculated in the global cohort and in subgroups of patients with and without HS-IR and risk of fibrosis. The ability of the combination to predict CVEs was analyzed using multivariable analysis after adjusting for the independent variables highlighted in section 2.5.

The association between independent variables and a diagnosis of CVE was studied using univariable and multivariable competing risk analysis (competing risk: death from non-cardiovascular causes). Factors with a *p* value < 0.1 and/or clinically relevant factors were included in the multivariable model.

For all tests, a 2-sided *p* value < 0.05 was considered statistically significant. The analysis was performed using SPSS, Version 29.0 (IBM Corp., Armonk, NY, USA).

## 3 Results

### 3.1 Study population

The study population comprised 7,286 PWH, with a follow-up of 39,124 person-years. At baseline, the median (IQR) age was 37 (30–45) years, 12.7% were female, 10.7% were in stage C3 according to the CDC classification, and the median nadir CD4 + lymphocyte count was 335/μL (200–471) (Table 1). The Supplementary material shows the baseline characteristics of the study population according to the values of the indices studied (TyG and FIB-4) (Supplementary material, A). Compared with individuals with normal values, those with TyG and/or FIB-4 above the cut-off had a median age of ≥36 years, were more severely ill before ART, and were more often HCV-infected. In addition, smoking, alcohol consumption, diabetes, hypertension, and higher cholesterol were more frequent. After correction for these factors, we are able to report the results set out below.

**TABLE 1:** Baseline characteristics: total values and values by subgroup*.

Variable		Total *N* = 7286	No CVEs *N* = 7149	CVEs or CV death *N* = 137	Non-CV death *N* = 204	*p*
Female sex		928 (12.7)	882 (12.7)	19 (13.9)	27 (13.2)	0.899
Age		36.9 (29.9−45.2)	36.5 (29.6−44.5)^a^	48.6 (39.6−58.3)b	48.3 (40−56.2)b	<0.001
Age >50		1070 (14.7)	917 (13.2)^a^	64 (46.7)^b^	89 (43.6)^b^	<0.001
Years with HIV infection		0.2 (0.1−1.1)	0.2 (0.1−1)	0.2 (0.1−2.6)	0.1 (0−2.8)	0.132
Route of infection	heterosexual; IDU; MSW; other	4930 (70); 263 (4); 1799 (26); 52 (1)	4790 (71); 213 (3); 1679 (25);^a^ 46 (1)	66 (50); 12 (9); 53 (40); 2 (2)^b^	74 (40); 38 (21); 67 (37); 4^b^ (2)	<0.001
Country of origin	Spain	3720 (51.1)	3494 (50.3)^a^	83 (60.6)^a^,^b^	143 (70.1)^b^	<0.001
	Europe	1393 (19.1)	1326 (19.1)^a^	32 (23.4)^a^	35 (17.2)^a^	
	Latin America & Caribbean	1612 (22.1)	1583 (22.8)^a^	14 (10.2)^b^	15 (7.4)^b^	
	North Africa	81 (1.1)	77 (1.1)^a^	2 (1.5)^a^	2 (1)^a^	
	Sub-Saharan Africa	275 (3.8)	266 (3.8)^a^	5 (3.6)^a^	4 (2)^a^	
	Other	57 (0.8)	54 (0.8)^a^	1 (0.7)^a^	2 (1)^a^	
	Unspecified	148 (2)	145 (2.1)^a^	0 (0)^a^	3 (1.5)^a^	
Educational level	Compulsory cycle	1952 (31.8)	1802 (30.8)^a^	63 (55.8)^b^	87 (54.7)^b^	<0.001
	Non-compulsory Secondary	2110 (34.4)	2037 (34.8)^a^	27 (23.9)^b^	46 (28.9)^a^,^b^	
	University/Higher	2067 (33.7)	2018 (34.5)^a^	23 (20.4)^b^	26 (16.4)^b^	
Nadir CD4 + cell count		335 (200−471)	341 (208−476)^a^	244 (100−417)^b^	166 (48−315)c	<0.001
Stage C3 AIDS		781 (10.7)	665 (9.6)^a^	27 (19.7)^b^	89 (43.6)c	<0.001
HCV coinfection		486 (6.8)	408 (6.0)^a^	23 (17.3)^b^	55 (27.6)^b^	<0.001
BMI		23.7 (21.6−26)	23.7 (21.6−26)^a^	25 (21.7−27.2)^a^,^b^	22.3 (20.1−26.9)^b^	0.028
FIB-4 index		0.93 (0.64−1.45)	0.91 (0.63−1.41)^a^	1.34 (0.88−2.52)^b^	1.65 (1.04−3.06)^b^	<0.001
TyG index		8.43 (8.08−8.78)	8.43 (8.07−8.78)^a^	8.68 (8.38−8.99)^b^	8.55 (8.25−8.98)^b^	<0.001
HS-IR		3629 (55.8)	3411 (55)^a^	103 (78.6)^b^	115 (70.1)^b^	<0.001
Risk of fibrosis (FIB-4 ≥ 1.3)		2199 (31.0)	2003 (29.6)^a^	68 (49.6)^b^	128 (65.0)c	<0.001
HS-IR/risk of fibrosis	No HS-IR or risk of fibrosis	2370 (32.5)	2327 (33.5)^a^	17 (12.4)^b^	26 (12.7)^b^	
	HS-IR or risk of fibrosis	4004 (55.0)	3822 (55.0)^a^	69 (50.4)^a^	113 (55.4)^a^	
	HS-IR and risk of fibrosis	912 (12.5)	796 (11.5)^a^	51 (37.2)^b^	65 (31.9)^b^	<0.001
Smokers (active and exsmokers <15 years)		2597 (54.2)	2457 (53.6)^a^	57 (72.2)^b^	83 (65.9)^b^	<0.001
High-risk alcohol intake**		125 (4.4)	106 (3.9)^a^	7 (17.1)^b^	12 (14.5)^b^	<0.001
Diabetes mellitus		185 (2.5)	155 (2.2)^a^	15 (10.9)^b^	15 (7.4)^b^	<0.001
	Treatment with OAD agents	52 (28.1)	44 (28.4)	5 (33.3)	3 (20.0)	0.706
	Treatment with insulin	23 (12.4)	20 (12.9)	3 (20.0)	0 (0.0)	0.229
Arterial hypertension***		1206 (24.3)	1128 (23.6)^a^	43 (55.8)^b^	35 (31.8)^a^	<0.001
Concomitant cardiovascular medication		251 (3.4)	230 (3.3)^a^	15 (10.9)^b^	6 (2.9)^b^	<0.001
Total cholesterol > 200 mg/dL		1037 (14.9)	986 (14.8)	27 (22.1)	24 (13.7)	0.074
CVRFs****	0	1068 (21.3)	1051 (22.0)^a^	4 (4.3)^b^	13 (10.2)^b^	<0.001
	1-2	3782 (75.5)	3600 (75.2)^a^	73 (78.5)^a^,^b^	109 (85.8)^b^	
	≥ 3	156 (3.1)	135 (2.8)^a^	16 (17.2)^b^	5 (3.9)^a^	
First ART	2NRTI + 1NNRTI	1732 (23.8)	1628 (23.4)^a^	51 (37.2)^b^	53 (26)^a^,^b^	<0.001
	2NRTI + 1IP	1213 (16.6)	1115 (16.1)^a^	33 (24.1)^b^	65 (31.9)^b^	
	2NRTI + 1II	3649 (50.1)	3536 (50.9)^a^	39 (28.5)^b^	74 (36.3)^b^	
	Other	692 (9.5)	666 (9.6)^a^	14 (10.2)^a^	12 (5.9)^a^	
Duration of first ART, months		18.2 (7.1−34.2)	18.6 (7.5−34.7)^a^	15.6 (4.3−33.4)^a^	5.3 (1.4−19)^b^	<0.001
Total months of follow-up (including time after event)		61.7 (30.5−97.0)	62.2 (31.1−97.1)^a^	95.4 (61.4-121.4)^b^	20.5 (3.9−54.6)c	<0.001
Months to event		60.4 (29.8−95.8)	62.2 (31.1−97.1)^a^	37.6 (15.5−79.0)^b^	20.5 (3.9−54.6)c	0.000

*Data are shown as median (IQR) or *n* (%). **Risky alcohol intake: Yes (≥17 standard per week if ♂ ≥28 standard per week if ♀) ***Arterial hypertension: Yes (SAP/DAP ≥ 140 and/or ≥90 mmHg and/or diagnosis of arterial hypertension and/or no beta-blockers) ****CVRFs evaluated: smoking, arterial hypertension, total cholesterol >200 mg/dL, diabetes mellitus, and cardiovascular medication a, b, c: Proportions differ significantly with previous column(s) (*p* < 0.05). MSM, men who have sex with men; IDU, intravenous drug use; BMI, body mass index; TyG, triglyceride-index; FIB-4, Fibrosis 4; HS-IR, hepatic steatosis-insulin resistance; OAD, oral antidiabetic; CVRF, cardiovascular risk factor; ART, antiretroviral therapy; IQR, interquartile range; SAP, systolic arterial pressure; DAP, diastolic arterial pressure.

### 3.2 Cardiovascular events

We recorded 168 CVEs in 137 patients and 204 non-cardiovascular deaths over a median follow-up period of 60.4 (95% CI, 59.0–62.0) months. We observed that, compared with non-CVE patients, those who developed CVEs during follow-up were older at initiation of ART. They were also characterized by a lower mean nadir CD4 + lymphocyte count, a higher percentage of AIDS diagnoses, and a higher percentage of heterosexual relations as the route of transmission. Regarding metabolic parameters, the percentage of patients with arterial hypertension and diabetes was significantly higher at baseline (i.e., before initiation of ART) in participants who developed CVEs (Table 1).

**FIGURE 2:**
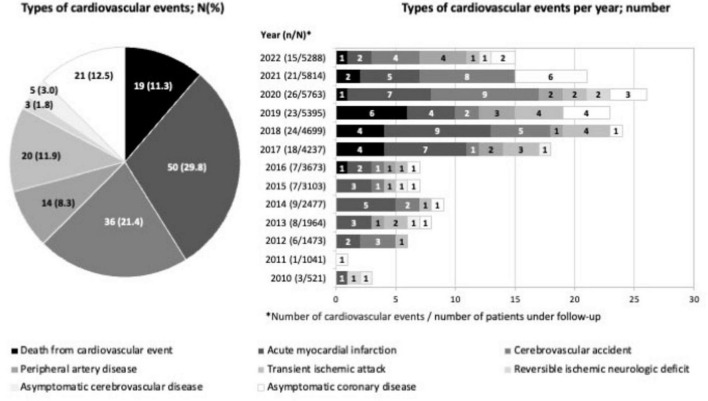
Cardiovascular events during follow-up shown by percentage and number. The most frequent CVEs during follow-up were acute myocardial infarction (29.8%) and stroke (21.4%). CVEs occurred after a median follow-up of 37.6 months.

Of the 168 CVEs that occurred during follow-up, acute myocardial infarction (29.8%) and stroke (21.4%) were the most frequent. A total of 19 deaths secondary to the event (11.3%) were recorded, along with 7 additional deaths after the event due to non-cardiovascular causes. CVEs occurred after a median follow-up of 37.6 months (95% CI, 28.4–57.7), with a significantly higher frequency from 2017 onward (128 vs. 40) (Figure 2).

We described the baseline characteristics of 204 patients who died of non-cardiovascular causes during follow-up. The patients in this subgroup were older, with more advanced HIV infection and a lower educational level (Table 1).

### 3.3 Impact of baseline TyG and FIB-4 on development of CVEs

**FIGURE 3:**
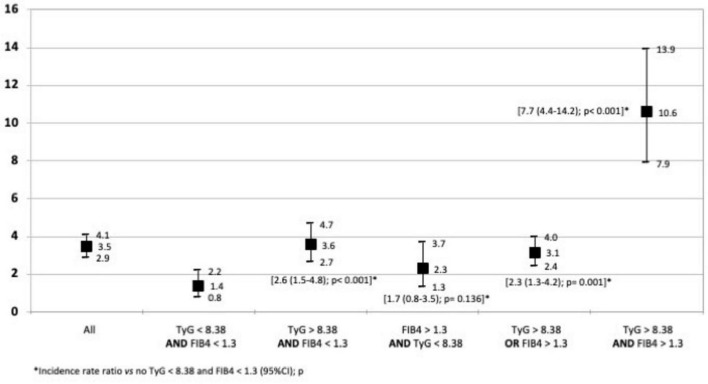
Global incidence of cardiovascular events in person-years with the 95% confidence interval. The figure shows the impact of baseline TyG and FIB-4 on development of CVEs. The differences in the incidence of CVEs with respect to baseline HS-IR and risk of fibrosis were significant. TyG > 8.38: Hepatic steatosis-insulin resistance FIB4 > 1.3: Risk of liver fibrosis.

The global incidence of CVEs was 3.5/1,000 person-years (95% CI, 2.9–4.1). At baseline, before initiating ART, we identified 3629 cases (55.8%) with HS-IR (TyG > 8.38) and 2199 cases (31%) with a risk of fibrosis (FIB-4 > 1.3). Both indices were elevated at baseline in 912 cases (12.5%) (Table 1). We found significant differences in the incidence of CVEs with respect to baseline HS-IR and risk of fibrosis. The incidence of CVEs in persons with and without HS-IR and risk of fibrosis was 10.6 (95% CI, 8.0–14.0) and 1.4 (95% CI, 0.8–2.2) per 1,000 person-years, respectively (*p* < 0.001). These values were 3.6 (95% CI, 2.7–4.7) for patients with TyG > 8.38 and FIB-4 < 1.3 and 3.1 (95% CI, 2.4-4.0), for patients with TyG > 8.38 or FIB-4 > 1.3, respectively (p < 0.001) (Figure 3).

**FIGURE 4:**
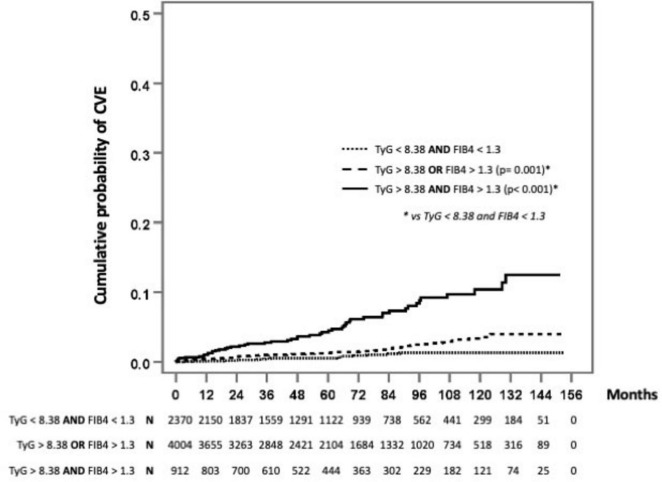
Probability of experiencing a cardiovascular event during follow-up. Survival analysis. The probability of experiencing a CVE during follow-up was significantly higher in PWH with elevated values in one index (TyG or FIB-4) or both indices than among those with normal indices. These differences were more pronounced after 60 months of follow-up. TyG > 8.38: Hepatic steatosis-insulin resistance. FIB4 > 1.3: Risk of liver fibrosis.

The probability of experiencing a CVE during follow-up was significantly higher in PWH with elevated values in one index (TyG or FIB-4) or both indices than among those with normal indices. These differences were more pronounced after 60 months of follow-up (Figure 4).

After adjusting for age, observation time, nadir CD4 + cell count, C3 AIDS stage, HCV coinfection, and number of cardiovascular risk factors, the only factor associated with CVEs was combination of HS-IR and risk of fibrosis (HR, 2.21; 95% CI, 1.2–4.1; *p* < 0.01), whereas HS-IR or risk of fibrosis was not (HR, 1.3; 95% CI, 0.75–2.27; *p* = 0.34) (Table 2).

**TABLE 2:** Competing risk regression analysis of factors associated with cardiovascular events.^a^

Variable	Univariable HR (95%CI); *p* value	Multivariable HR (95%CI); *p* value
Age	1.08 (1.06−1.09); <0.001	1.06 (1.04−1.07); <0.001
Female sex	1.1 (0.68−1.78); 0.71	—
Years with HIV infection	1.04 (1.01−1.08); 0.018	1.01 (0.98−1.05); 0.4
Nadir CD4 + cell count <200	2.22 (1.58−3.12); <0.001	1.5 (1−2.24); 0.051
Stage 3 AIDS	2.02 (1.32−3.08); 0.001	1.04 (0.64−1.7); 0.88
HCV	2.4 (1.53−3.75); <0.001	1.66 (0.98−2.8); 0.061
**CVRF^b^**		
1−2 CVRFs vs. none	4.5 (1.64−12.3); 0.004	3.17 (1.16−8.641); 0.024
≥3 CVRFs vs. none	24.9 (8.3−75); <0.001	8.93 (2.86−27.9); <0.001
TyG > 8.38	2.84 (1.87−4.31); <0.001	—
FIB4 > 1.3	2.01 (1.44−2.81); <0.001	—
TyG > 8.38 OR FIB4 > 1.3	2.27 (1.33−3.87); 0.003	1.3 (0.75−2.27); 0.34
TyG > 8.38 AND FIB4 > 1.3	7.34 (4.24−12.7); <0.001	2.21 (1.2−4.1); 0.01

^a^Competing risk: non-CV death

^b^CVRF evaluated: smoking, AHT, TC > 200, DM, and cardiovascular medication.

A sensitivity analysis restricted to the most severe CVE events, defined as fatal or non-fatal myocardial infarction or stroke revealed the same results as in the analysis of all the CVEs (Supplementary material, B).

## 4 Discussion

Our study highlights the association between HS-IR and risk of fibrosis and the increased risk of CVEs in PWH. We found that the prevalence of hepatic steatosis was higher than 50% and that the risk of hepatic fibrosis was higher than 30% at diagnosis of HIV, before initiation of ART, thus highlighting the importance of the infection itself in these conditions. Moreover, PWH with TyG > 8.38 and FIB-4 > 1.3 at diagnosis of HIV had more than double the risk of a CVE during follow-up. This risk was independent of classic cardiovascular risk factors, age, AIDS stage, and time with HIV. Therefore, the markers assessed are of considerable use in clinical practice, in addition to being inexpensive and easily applied in the diagnosis of HIV.

Our results are consistent with those of previous studies emphasizing the heightened cardiovascular risk in PWH, although we also contribute novel insights. We observed an incidence rate of 3.5 CVEs per 1,000 person-years, consistent with prior findings that underscore the heightened cardiovascular risk in this population (1). Furthermore, individuals with both HS-IR and a risk of fibrosis exhibited a significantly higher incidence of CVEs (10.6 per 1,000 person-years). Interestingly, in a European cohort of non-PWH, Martinez-Urbistondo et al. (16) demonstrated that the combined presence of elevated FIB-4 (suggesting liver fibrosis) and TyG (reflecting IR) significantly increased the risk of major adverse cardiovascular events, independently of classic cardiovascular risk factors. Similarly, in a Chinese cohort of PWH, Luo et al. (22) identified a significant association between higher baseline TyG index and risk of cardiovascular disease, despite not evaluating liver fibrosis. These findings strengthen evidence that a combination of both metabolic and hepatic markers provides a more comprehensive assessment of cardiovascular risk in both PWH and non-PWH.

Wong et al. (3) investigated cardiovascular outcomes in veterans with MASLD and HIV, finding a higher incidence of major adverse cardiovascular events than in patients with MASLD alone. This suggests that HIV itself would exacerbate cardiovascular risk in patients with concurrent metabolic liver disease. Furthermore, a meta-analysis by Zhu et al. (23) confirmed an elevated cardiovascular risk in PWH, with a higher prevalence of dyslipidemia, coronary artery disease, and cerebrovascular events than in the general population. In our study, we evaluated cardiovascular risk as a combination of hypertension, smoking, dyslipidemia, and diabetes and found that only 20% of patients did not have a cardiovascular risk factor, with most patients having 1 or 2 risk factors. Therefore, we categorized the number of risk factors as 1-2 vs. 3 or more to enable adjustment in the multivariable analysis. Our findings demonstrated that, while these factors play a significant role in the occurrence of CVE, the presence of HS-IR with risk of fibrosis independently added a more than 2-fold increased risk. These findings underscore the need to adapt cardiovascular risk prediction models to better reflect the unique metabolic challenges faced by PWH.

Recent studies have explored the prevalence of hepatic steatosis and fibrosis in PWH receiving stable ART and diagnosed using transient elastography (24–26). The authors report high prevalence, especially in persons with overweight and obesity and in those with DM2. Lin et al. (24) found that MASLD was independently associated with an altered advanced lipoprotein profile and an increase in the levels of the metabolites associated with insulin resistance in the liver, pointing to a greater cardiovascular risk in this population. Furthermore, van Eekeren et al. (25) performed a separate analysis of lean PWH with MASLD and found that this subgroup seems to have a greater incidence of CVEs and metabolomic and lipoproteomic perturbations. In our opinion, the results of these studies, which show potential mechanisms underlying the increased risk of CVEs in PWLH and MASLD, highlight the importance of identifying affected patients early. Of those participants who developed CVEs based on IR/FIB-4 only one third of patients had overweight or obesity and 2.5% had diabetes, thus highlighting the importance of other, underlying mechanisms that are independent of the classical risk factors for MASLD. Consequently, the universally available TyG and FIB-4 indices gain considerable relevance owing to their easy applicability and low cost compared with transient elastography. Of note, the TyG index has also been widely used to identify persons with insulin resistance, making it doubly useful.

An important distinction of our study is the relatively young age of the cohort (mean age, 37 years) compared to the older populations analyzed in previous studies, such as the veteran cohort in the study by Wong et al. (3) and the European cohort in Martinez-Urbistondo et al. (16). Despite their young age, we found that this population had a high prevalence of cardiovascular risk factors and MASLD. This observation suggests that metabolic and hepatic markers may play a crucial role in identifying cardiovascular risk at earlier stages, even in younger populations. The impact of age on cardiovascular risk stratification warrants further investigation, as younger individuals with HS-IR and risk of fibrosis may represent a particularly high-risk group requiring earlier intervention strategies. In line with this, the CARDIA study by Xu et al. (26) demonstrated that a high TyG index in young adulthood is associated with an increased risk of cardiovascular disease and mortality in later life, thus emphasizing the long-term prognostic value of this marker. The probability of experiencing a CVE during follow-up was significantly higher in PWH with elevated values in one index (TyG or FIB-4) or both indices than among those with normal indices. These differences were more pronounced after 60 months of follow-up. Therefore, evaluation of HS-IR and risk of fibrosis based on TyG and FIB-4 in clinical practice constitutes a cost-effective approach that could enhance cardiovascular risk stratification and complement further research in this field. Moreover, these indices are easily derived from standard laboratory tests, making them feasible for widespread implementation in diverse healthcare settings, including resource-limited environments, where advanced cardiovascular risk assessments may not be readily available. By identifying high-risk individuals using TyG and FIB-4, targeted interventions could be implemented to mitigate cardiovascular risk. These would include lifestyle modifications such as personalized dietary and exercise interventions aimed at improving metabolic health. Pharmacological strategies, including the use of insulin-sensitizing agents (e.g., metformin) and lipid-lowering therapies (e.g., statins), could be considered for persons with persistently elevated risk markers. Furthermore, in individuals with evidence of hepatic fibrosis, hepatoprotective strategies such as weight management, avoidance of hepatotoxic agents, and potential antifibrotic therapies may be warranted.

### 4.1 Study limitations

Despite the strengths of our study, certain limitations should be acknowledged. First, while TyG and FIB-4 are widely used as surrogate markers, their accuracy and predictive value may vary across different subgroups of PWH, particularly those with coexisting metabolic conditions or differing levels of liver disease progression. Additionally, our study population may not fully represent the broader PWH community, thus limiting the generalizability of our findings. Notably, our cohort included a relatively low proportion of women, which may influence the applicability of our findings to female PWH. This is particularly relevant given that Luo et al. (22) found sex-specific differences in the association between the TyG index and cardiovascular disease, namely, a potentially greater predictive value in women. Future studies should explore sex-based differences further to ensure more tailored risk assessment strategies.

Another potential limitation is the observational nature of our study, which precludes causal inference. While our results suggest a strong association between these biomarkers and cardiovascular risk, unmeasured confounders such as diet, physical activity, and genetic predisposition could have influenced outcomes. Furthermore, the use of non-invasive surrogate markers rather than liver biopsy or imaging studies may introduce some degree of misclassification in the assessment of fibrosis.

A relevant baseline imbalance in cardiovascular risk factors remained only partially corrected, since covariates were not analyzed separately. This raises the possibility of residual confounding. Moreover, the absence of data to contrast our findings with validated prediction models (e.g., SCORE2) precludes determination of their added predictive value. Nevertheless, the persistence of the adjusted HR (2.2) for the combination of elevated TyG and FIB-4 highlights the need for further research, particularly given the feasibility of incorporating these markers in routine practice.

Furthermore, additional metabolic and inflammatory biomarkers should be explored to further refine cardiovascular risk stratification in PWH. Integrating longitudinal assessments of HS-IR and risk of fibrosis may also provide more dynamic insights into progression of cardiovascular risk. Our data provide the rationale for follow-up studies to assess whether current risk prediction based on classical cardiovascular risk factors can be enhanced by incorporating indices such as these in PWH.

## 5 Conclusion

Our study supports the growing body of evidence that HS, IR, and liver fibrosis increase cardiovascular risk in PWH. Incorporating markers such as TyG and FIB-4 into clinical practice could enhance risk stratification, enabling early interventions to mitigate cardiovascular morbidity and mortality in this vulnerable population.

## Data Availability

The raw data supporting the conclusions of this article will be made available by the authors, without undue reservation.

## Footnotes

1
https://hicdep.org/Wiki/v/12/pt/2

## References

[1] Shah A Stelzle D Lee K Beck E Alam S Clifford S Global burden of atherosclerotic cardiovascular disease in people living with the human immunodeficiency virus. *Circulation.* (2018) 138:1100–12. 10.1161/CIRCULATIONAHA.117.033369 29967196 PMC6221183

[2] Bertrand L Velichkovska M Toborek M. Cerebrovascular toxicity of antiretroviral therapy. *J Neuroimmune Pharmacol.* (2021) 16:74–89. 10.1007/s11481-019-09858-x 31209776 PMC7952282

[3] Wong R Yang Z Yeoh A Do A Ahmed A Cheung R. Impact of HIV infection on liver and cardiovascular outcomes in veterans with metabolic dysfunction-associated steatotic liver disease. *Am J Gasteroenterol.* (2024) 119:1841–8. 10.14309/ajg.0000000000002760 38477465

[4] Michel M Labenz C Wahl A Anders M Armandi A Huber Y Prevalence and risk factors of nonalcoholic steatohepatitis with significant fibrosis in people with HIV. *AIDS.* (2022) 36:1665–74. 10.1097/QAD.0000000000003312 35849074 PMC9451864

[5] Montes M Busca C Rava M Bernardino J Rivero A Martín-Carbonero L Hepatic steatosis-insulin resistance and type 2 diabetes in people with HIV at diagnosis: effect of initial antiretroviral therapy. *AIDS.* (2024) 38:1982–7. 10.1097/QAD.0000000000004008 39474653

[6] Heseltine T Murray S Ortega-Martorell S Oliver I Lip G Khoo S. Associations of hepatosteatosis with cardiovascular disease in hiv-positive and hiv-negative patients: the liverpool HIV-heart project. *J Acquir Immune Defic Syndr.* (2021) 87:1221–7. 10.1097/QAI.0000000000002721 33990492

[7] Falutz J. HIV infection, body composition changes and related metabolic complications: contributing factors and evolving management strategies. *Curr Opin Clin Nutr Metab Care.* (2011) 14:255–60. 10.1097/MCO.0b013e3283457a8f 21460720

[8] Torgersen J So-Armah K Freiberg M Goetz M Budoff M Lim J Comparison of the prevalence, severity, and risk factors for hepatic steatosis in HIV-infected and uninfected people. *BMC Gastroenterology.* (2019) 19:52. 10.1186/s12876-019-0969-1 30987601 PMC6466708

[9] Unger G Benozzi S Perruzza F Pennacchiotti G. Triglycerides and glucose index: a useful indicator of insulin resistance. *Endocrinol Nutr.* (2014) 61:533–40. 10.1016/j.endonu.2014.06.009 25174769

[10] Guerrero-Romero F Simental-Mendía L González-Ortiz M Martínez-Abundis E Ramos-Zavala M Hernández-González S The product of triglycerides and glucose, a simple measure of insulin sensitivity. Comparison with the euglycemic-hyperinsulinemic clamp. *J Clin Endocrinol Metab.* (2010) 95:3347–51. 10.1210/jc.2010-0288 20484475

[11] Fedchuk L Nascimbeni F Pais R Charlotte F Housset C Ratziu V Performance and limitations of steatosis biomarkers in patients with nonalcoholic fatty liver disease. *Aliment Pharmacol Ther.* (2014) 40:1209–22. 10.1111/apt.12963 25267215

[12] Busca C Sánchez-Conde M Rico M Rosas M Valencia E Moreno A Assessment of noninvasive markers of steatosis and liver fibrosis in human immunodeficiency virus-monoinfected patients on stable antiretroviral regimens. *Open Forum Infect Dis.* (2022) 9:ofac279. 10.1093/ofid/ofac279 35873289 PMC9297309

[13] Zhang S Du T Zhang J Lu H Lin X Xie J The triglyceride and glucose index (TyG) is an effective biomarker to identify nonalcoholic fatty liver disease. *Lipids Health Dis.* (2017) 16:15. 10.1186/s12944-017-0409-6 28103934 PMC5248473

[14] Lemoine M Assoumou L De Wit S Girard P Valantin M Katlama C Diagnostic accuracy of noninvasive markers of steatosis, NASH, and liver fibrosis in HIV-monoinfected individuals at risk of nonalcoholic fatty liver disease (NAFLD): results from the ECHAM study. *J Acquir Immune Defic Synd.* (2019) 80:e86–94. 10.1097/QAI.0000000000001936 30570529

[15] So-Armah K Lim J Lo Re V Tate JP Chang C-CH Butt AA FIB-4 stage of liver fibrosis is associated with incident heart failure with preserved, but not reduced, ejection fraction among people with and without HIV or hepatitis C. *Prog Cardiovasc Dis.* (2020) 63:184–91. 10.1016/j.pcad.2020.02.010 32068085 PMC7278895

[16] Martinez-Urbistondo D D’Avola D Navarro-González D Sanchez-Iñigo L Fernandez-Montero A Perez-Dias-Del-Campo N Interactive role of surrogate liver fibrosis assessment and insulin resistance on the incidence of major cardiovascular events. *J Clin Med.* (2022) 11:5190. 10.3390/jcm11175190 36079118 PMC9456724

[17] Feinstein M Hsue P Benjamin L Bloomfield G Currier J Freiberg M Characteristics, prevention, and management of cardiovascular disease in people living with HIV: a scientific statement from the American heart association. *Circulation.* (2019) 140:e98–124. 10.1161/CIR.0000000000000695 31154814 PMC7993364

[18] Sobrino-Vegas P Gutiérrez F Berenguer J Labarga P García F Alejos-Ferreras B [The cohort of the Spanish HIV research network (CoRIS) and its associated biobank; organizational issues, main findings and losses to follow-up]. *Enferm Infecc Microbiol Clin.* (2011) 29:645–53. 10.1016/j.eimc.2011.06.002 21820763

[19] World Health Organization. Implementation of the international statistical classification of diseases and related health problems, tenth revision (ICD-10). *Epidemiol Bull.* (1997) 18:1–4. 9197082

[20] Sterling R Lissen E Clumeck N Sola R Correa M Montaner J Development of a simple noninvasive index to predict significant fibrosis in patients with HIV/HCV coinfection. *Hepatology.* (2006) 43:1317–25. 10.1002/hep.21178 16729309

[21] Ministerio de Sanidad. *Spanish ministry of health criteria for alcohol consumption.* (2025). Available online at: https://pnsd.sanidad.gob.es/ciudadanos/informacion/alcohol/menuAlcohol/largoPlazo.htm (accessed May 16, 2025).

[22] Luo Y Sun L He Y Zhao F Shan D Bu F The triglyceride-glucose index trajectories are associated with cardiovascular diseases in people living with HIV: evidence from a prospective cohort study in china. *BMC Public Health.* (2025) 25:465. 10.1186/s12889-025-21744-1 39910507 PMC11800576

[23] Zhu S Wang W He J Duan W Ma X Guan H Higher cardiovascular disease risks in people living with HIV: a systematic review and meta-analysis. *J Glob Health.* (2024) 14:04078. 10.7189/jogh.14.04078 38666515 PMC11046517

[24] Lin K Vilar-Gomez E Corey K Connelly M Gupta S Lake J MASLD in persons with HIV is associated with high cardiometabolic risk as evidenced by altered advanced lipoprotein profiles and targeted metabolomics. *Lipids Health Dis.* (2024) 23:339. 10.1186/s12944-024-02317-4 39420356 PMC11484191

[25] van Eekeren L Vadaq N Blaauw M Groenendijk A Vos J Nelwan E Distinct metabolic perturbations link liver steatosis and incident CVD in lean but not obese PWH. *BMC Med.* (2025) 23:78. 10.1186/s12916-025-03914-5 39934780 PMC11817758

[26] Xu X Huang R Lin Y Guo Y Xiong Z Zhong X High triglyceride-glucose index in young adulthood is associated with incident cardiovascular disease and mortality in later life: insight from the CARDIA study. *Cardiovasc Diabetol.* (2022) 21:155. 10.1186/s12933-022-01593-7 35962377 PMC9375240

