# Supplementary material for: Triglyceride–glucose index and FIB-4 score in relation to cardiovascular disease risk among people with HIV: a retrospective cohort study
Source: Front Med (Lausanne). 2025 Nov 6;12:1638071. doi: 10.3389/fmed.2025.1638071 (PMC12631423; doi:10.3389/fmed.2025.1638071)
Supplement: Supplementary file 1 [file Data_Sheet_1.pdf]

Supplementary material

A) Analysis of the baseline characteristics of the study population according to values of TyG and FIB-4.

	**TyG < 8.38 AND FIB4 < 1.3 (N= 2370)**	**TyG > 8.38 OR FIB4 > 1.3 (N= 4004)**	**TyG > 8.38 AND FIB4 > 1.3 (N= 912)**	**p**
Female sex	286 (12.1)	506 (12.6)	136 (14.9)	0.087
Age	31.8 (26.8-38) [a]	38.4 (31.3-45.7) [b]	47 (39.4-54.6) [c]	<0.001
Years with HIV infection	0.2 (0.1-0.9) [a]	0.2 (0.1-1.3) [a]	0.1 (0-0.8) [b]	<0.001
Nadir CD4+ cell count	380 (268-510) [a]	325 (189-460) [b]	229 (80-391) [c]	<0.001
Stage C3 AIDS	134 (5.7) [a]	540 (13.5) [b]	219 (24) [c]	<0.001
HCV coinfection	408 (6.0) [a]	23 (17.3) [b]	55 (27.6) [b]	<0.001
BMI	23.1 (21.2-25) [a]	24.1 (22-26.4) [b]	24.3 (22-26.6) [b]	<0.001
FIB-4 index	0.72 (0.55-0.95) [a]	0.98 (0.66-1.49) [b]	1.84 (1.51-2.71) [c]	<0.001
TyG index	8.04 (7.83-8.21) [a]	8.67 (8.46-8.96) [b]	8.78 (8.59-9.13) [c]	<0.001
Smokers (active and exsmokers <15 years)	802 (50.3) [a]	1468 (55.9) [b]	327 (57.5) [c]	<0.001
High-risk alcohol intake**	13 (1.5) [a]	89 (5.6) [b]	23 (6.3) [b]	<0.001
Diabetes mellitus	14 (0.6) [a]	97 (2.4) [b]	74 (8.1) [c]	<0.001
Arterial hypertension***	274 (17.3) [a]	713 (25.7) [b]	219 (36.1) [c]	<0.001
Concomitant cardiovascular medication	19 (0.8) [a]	146 (3.6) [b]	86 (9.4) [c]	<0.001
Total cholesterol > 200 mg/dL	173 (7.6) [a]	713 (18.7) [a]	151 (17.6) [b]	<0.001
Lipid-lowering	3 (0.1) [a]	45 (1.1) [b]	32 (3.5) [c]	<0.001
CVRFs**** 0	465 (30.1) [a]	526 (18.7) [b]	77 (11.9) [c]	
1-2	1063 (68.8) [a]	2193 (78) [b]	526 (81) [b]	
≥3	16 (1) [a]	94 (3.3) [b]	46 (7.1) [c]	<0.001
First ART 2NRTI+1NNRTI	594 (25.1) [a]	962 (24) [a]	176 (19.3) [b]	
2NRTI+1IP	326 (13.8) [a]	694 (17.3) [b]	193 (21.2) [c]	
2NRTI+1II	1172 (49.5) [a]	2002 (50) [a]	475 (52.1) [a]	
Other	278 (11.7) [a]	346 (8.6) [b]	68 (7.5) [b]	<0.001
Duration of first ART, months	18.4 (8.1-35) [a]	18.2 (6.9-34.4) [a,b]	17 (5.9-32.5) [b]	0.015
Total months of follow-up (including time after event)	55.3 (27-93.9) [a]	63.7 (32.4-97.6) [b]	61.6 (28.1-100.4) [a,b]	0.002
Months to event	54.9 (26.8-93.5) [a]	62.9 (31.7-96.6) [b]	57.7 (25.6-96.3) [a,b]	0.003

B) Subanalysis of patients with myocardial infarction, stroke, and/or cardiovascular death.

N= 94 patients (1.3% [95% CI: 1-1.6])

	No event		Event		Death (CV)		
	N	%	N	%	N	%	p
TyG <= 8.38 AND FIB4 <= 1.3	2333	33.4	10	10.6	27	13.1	
TyG > 8.38 AND/OR FIB4 <= 1.3	3839	55.0	51	54.3	114	55.3	
TyG >= 8.38 AND FIB4 > 1.3	814	11.7	33	35.1	65	31.6	
ALL	6986	100	94	100	206	100	<0.001

Survival analysis.

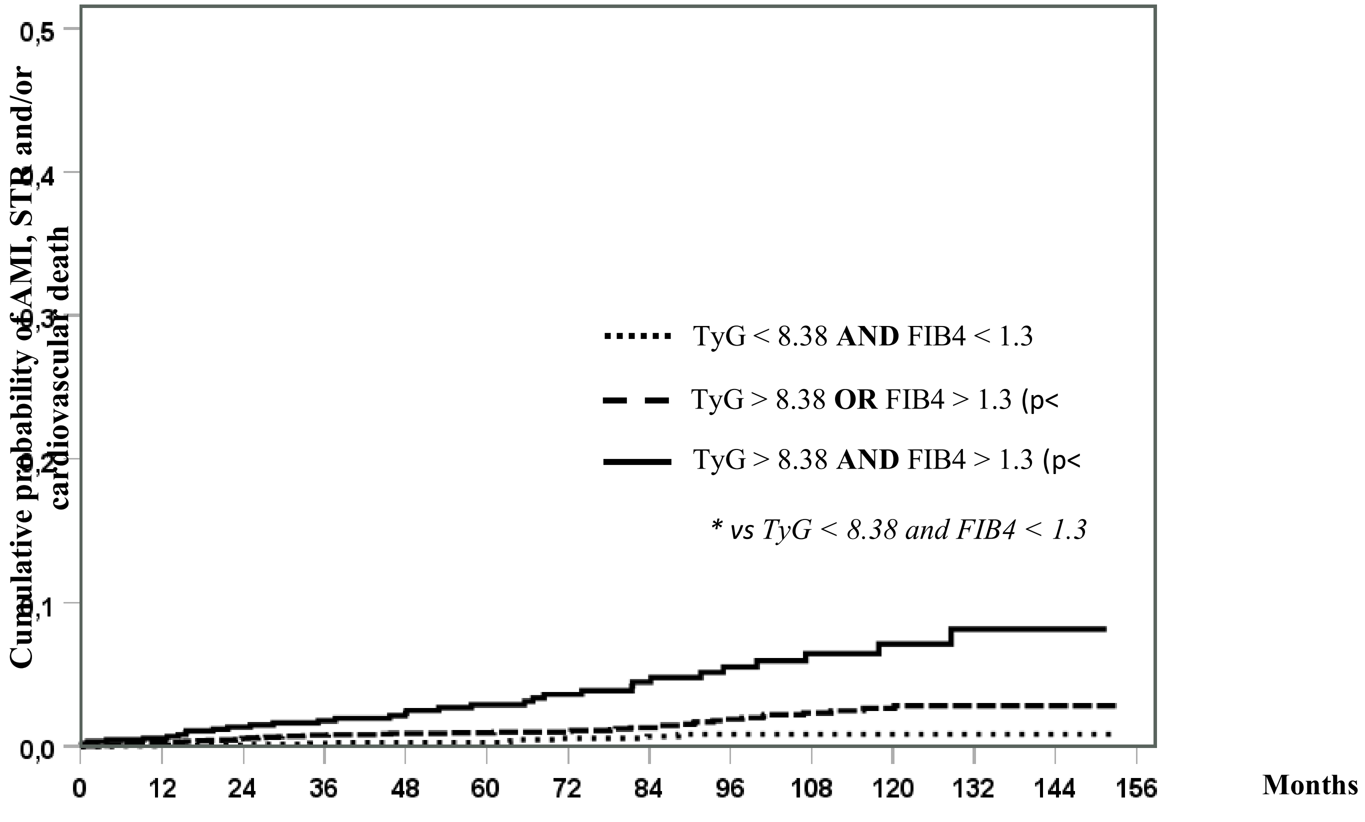

**Competing risk regression analysis of factors associated with cardiovascular events.** [a]	**Multivariable** HR (95%CI); p value
Age	1.05 (1.03-1.07); <0.001
Years with HIV infection	1.02 (0.98-1.06); 0.27
Nadir CD4+ cell count <200	1.42 (0.87-2.3); 0.16
Stage 3 AIDS	1.16 (0.66-2.04); 0.6
HCV	1.62 (0.88-3); 0.12
CVRF[b]	
1-2 CVRFs vs none	2.91 (0.92-9.23); 0.07
≥ 3 CVRFs vs none	9.6 (2.62-35.17); 0.001
TyG > 8.38 or FIB4 > 1.3	1.73 (0.84-3.54); 0.13
TyG > 8.38 and FIB4 > 1.3	**2.68 (1.2-6); 0.016**

[a]Competing risk: non-CV death

[b]CVRF evaluated: smoking, AHT, TC > 200, DM, and cardiovascular medication
